# Mirabegron attenuates porcine ureteral contractility via α1-adrenoceptor antagonism

**DOI:** 10.1007/s00210-022-02244-0

**Published:** 2022-04-21

**Authors:** Iris Lim, Russ Chess-Williams

**Affiliations:** Centre for Urology Research, Faculty of Health Science & Medicine, Bond University, Robina, QLD 4229 Australia

**Keywords:** α_1_-adrenoceptor, Porcine, Smooth muscle contraction, Ureter, β-adrenoceptor, Mirabegron

## Abstract

The β_3_-agonist mirabegron is thought to induce relaxation of the detrusor muscle, contributing to the improvement of overactive bladder symptoms. There has been recent interest in purposing mirabegron as a medical expulsive therapy drug to improve the passage of smaller kidney stones by relaxing the ureteral smooth muscles. The aim of this study was to determine the effects of mirabegron on the activity of the ureter. Additionally, we investigated the receptor and mechanisms through which mirabegron exerts these effects. In vitro agonist-induced responses of isolated porcine distal ureteral tissues were measured in the absence and presence of mirabegron in organ bath experiments. The responses were expressed as frequency, area under the curve and maximum amplitude. Mirabegron at concentrations of 100 nM and lower failed to suppress phenylephrine- or 5-HT-induced contractions in the porcine ureteral strip. Mirabegron at 1 μM and 10 μM produced a rightward shift of phenylephrine concentration–response curves in these tissues. This effect of mirabegron (10 μM) was not present in 5-HT concentration–response curves. The mirabegron effect on phenylephrine-induced contractions was also not abolished by β-adrenoceptor antagonist SR 59230A (10 μM), β-adrenoceptor antagonist propranolol (10 μM), α_2_-adrenoceptor antagonist yohimbine (30 nM), and nitric oxide synthase inhibitor l-NNA (10 μM). The present results show that mirabegron suppresses ureteral contractile responses in the porcine ureter via α_1_-adrenoceptor antagonism, since their effects were not present when the tissues were contracted with 5-HT. Furthermore, the inhibitory effects by mirabegron were not affected by β_3_-adrenoceptor antagonists.

## Introduction

Urolithiasis, or known as urinary stones, is a widespread condition worldwide affecting up to 10% of the population, with its healthcare burden cost and prevalence continuing to increase (Gillams et al. 2021). While stones that are smaller in size can usually pass to the bladder spontaneously with urine, larger stones might lodge in the ureter. This usually results in a pressing pain, known as ureteral or renal colic, which is frequently presented clinically in patients. Ureteral colic is proposed to be induced by elevated intraureteral pressure which is caused by the constriction of the ureteral smooth muscle wall (Campschroer et al. 2018). In cases of smaller stones, medical expulsive therapy can be recommended with the aim of promoting stone passage by reducing constriction and relaxing the ureteral tube. Since the introduction of medical expulsive therapy, tamsulosin, the α_1_-adrenoceptor antagonist is the most commonly used drug and continues to be the recommendation of most urological associations to improve small stone passage (Assimos et al. 2016). This is not unexpected, as the main innervation of the ureter is controlled by the adrenergic system where the net effect of noradrenaline application results in contraction of the smooth muscles via α_1_-adrenoceptor stimulation (Forman et al. 1978).

The use of a β_3_-adrenoceptor agonist mirabegron for the management of overactive bladder has been shown to be effective in the recent years, reducing both urinary frequency and urgency, with low incidence of side effects and thus, high tolerability profile (Takahashi et al. 2022). Mirabegron is proposed to function by activating the β_3_-adrenoceptors in the human detrusor smooth muscle, inducing a relaxation during the storage phase of micturition (Svalo et al. 2013; Takasu et al. 2007). Previous studies have also shown that mirabegron is capable of relaxing other smooth muscle tissues of the urinary tract like the urethra and the prostate (Alexandre et al. 2016; Huang et al. 2021). Given the proximity of the ureter, particularly the distal section, to these organs, there has been recent interest in purposing mirabegron as a medical expulsive therapy alternative to tamsulosin to promote stone passage (Tang et al. 2021). This study aimed to investigate the effects of mirabegron on ureteral contractions in isolated porcine tissues. This animal model has been shown in a previous study to be a reliable pharmacological and physiological model, similar to that of the healthy human ureter (Lim et al. 2020). Additionally, this study also aimed to investigate the mechanisms through which mirabegron exerts its effects on this tissue.

## Materials and methods

### Tissue specimen origin and preparation

The urinary bladders (with ureters and urethra attached) of 6-month-old female Landrace pigs were obtained from a local abattoir and placed in ice-cold Krebs-bicarbonate solution (4 °C) composed of NaCl (118.4 mM), KCl (4.7 mM), NaHCO_3_ (24.9 mM), glucose (11.7 mM), CaCl_2_ (1.9 mM), MgSO_4_ (1.2 mM) and KH_2_PO_4_ (1.2 mM) and immediately transported to the laboratory within 2 h. The distal segment of the ureter was isolated and dissected into 4 mm long tube sections. These rings were cut opened (where the full diameter measures 6–7 mm) and suspended longitudinally. Paired tissues strips were utilised in the experiments of this study where adjacent tube sections of the same ureter were set up in separate organ baths and considered to be ‘paired’. The distal ureter was examined, as this is the most common site for urinary stone lodgement clinically (El-Barky et al. 2014). The urothelium and lamina propria were left intact with the smooth muscle strips in all experiments.

The tissue strips were mounted longitudinally under approximately 1.5 g tension in 8 ml EZ-Bath organ baths (Global Towns Microtechnology, Sarasota, FL, USA) containing Krebs-bicarbonate solution. The baths were maintained at 37 °C and continuously gassed with 95% O_2_ and 5% CO_2_ at pH 7.4. Equilibration of tissue strips was performed for 1 h with fresh solution washouts every 15 min, before addition of any drug. The isometric tension developed by the tissues was recorded via a Powerlab recording system and the LabChart software (ADInstruments, Castle Hill, NSW, Australia).

### Relaxatory effects of mirabegron on pre-contracted ureter

To investigate potential relaxatory effects of β_3_-adrenoceptor agonist mirabegron, paired tissue strips were first pre-contracted with α_1_-adrenoceptor agonist phenylephrine (300 μM) or 5-hydroxytryptamine (5-HT) (100 μM). Upon generation of a consistent pattern of contractile activity (approximately 10 min), mirabegron was cumulatively added to one tissue strip (100 nM–1 mM), while the other strip acted as a vehicle and time control. These experiments were then repeated in the presence of β-adrenoceptor antagonist SR 59230A (10 μM).

### Effects of mirabegron on agonist concentration–response

To determine the effects of mirabegron on phenylephrine and 5-HT-induced concentration–response, paired tissues strips were set up. Mirabegron (100 nM, 1 μM or 10 µM) was added to one tissue strip, while the other acted as a vehicle control. Following a 30-min incubation, tissue response to increasing concentrations of either phenylephrine (100 nM–1 mM) or 5-HT (300 nM–300 μM) was measured.

### Mechanisms of mirabegron-induced effects

In this part of the study, we aimed to investigate the receptor and/or mechanism through which mirabegron mediates its effects on the phenylephrine responses in the distal ureteral tissue. Pairing tissue strips from the same ureter, we incubated all tissues with either β-adrenoceptor antagonist SR 59230A (10 μM), β_1_- and β_2_-adrenoceptor blocker propranolol (10 μM), nitric oxide synthase inhibitor l-NNA (10 μM) or α_2_-adrenoceptor antagonist yohimbine (30 nM) for 30 min. Simultaneously, one of the paired strips was also incubated with mirabegron (10 μM) and concentration–response curves to phenylephrine were then performed.

### Data and statistical analysis

**Fig. 1:**
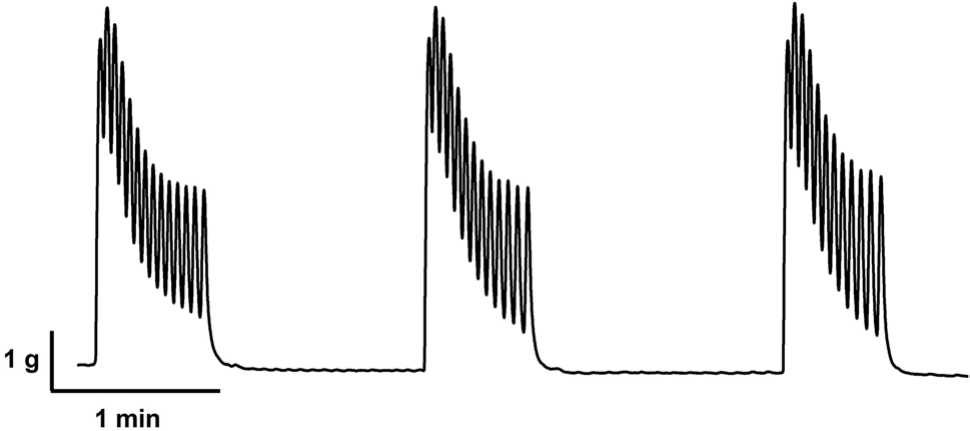
Raw data trace showing bursts of phasic contractions developed by the isolated porcine distal ureter after a single addition of phenylephrine (300 μM)

In response to phenylephrine or 5-HT, isolated ureteral strips developed bursts of phasic contractile activity (Fig. 1). These contractile responses appeared to increase in frequency (contractions per minute) in a concentration-dependent manner. The amplitude of the phasic contractions did not appear to have an observable pattern in this study. Area under the curve (AUC) by weight was calculated to determine the overall contractile responses of the ureter, accounting for changes in both amplitude and frequency of contractions. The frequency and AUC were measured over 5 min for each variable. The highest amplitude of the phasic contractions by tissue weight was also assessed.

The GraphPad Prism software (GraphPad, San Diego, CA, USA) was used to perform all statistical analysis and graphical representation. All data were expressed as mean ± SD of ‘*n*’ preparations, where ‘*n*’ is the number of animals. For each experiment, a sample size of *n* = 8 was used. However, occasionally, some tissues strips were not responsive or minimally responsive and these were considered outliers. This study is deemed to be of an explorative nature rather than a hypothesis-design study (Michel et al. 2020). pEC_50_ values were calculated as the negative logarithm of the agonist concentration required to achieve 50% of maximal contractile response. Concentration–response curves were compared using two-way ANOVA, while pEC_50_ and maximum amplitude values, using two-tailed, paired Student’s *t* tests, where *p* < 0.05 was considered statistically significant.

### Drugs and chemicals used

The chemicals used for the Krebs-bicarbonate solution were of analytical grade and purchased from Sigma-Aldrich (Castle Hill, NSW, Australia). (R)-( −)-Phenylephrine hydrochloride, 1-(2-Ethylphenoxy)-3-[[(1S)-1,2,3,4-tetrahydro-1-naphthalenyl]amino]-(2S)-2-propanol hydrochloride (SR 59230A), propranolol hydrochloride, Nω-Nitro-L-arginine (l-NNA) and yohimbine hydrochloride were obtained from Sigma-Aldrich (Castle Hill, NSW, Australia). Serotonin hydrochloride (5-HT) was obtained from Abcam (Melbourne, VIC, Australia) and mirabegron from Tocris (Noble Park, Victoria, Australia). All drugs were dissolved in distilled H_2_O except mirabegron and yohimbine which were dissolved in DMSO (final concentration of DMSO never exceeded 0.3%). All experiments were performed with a vehicle control where an equivalent amount of solvent (distilled H_2_O or DMSO) was added into the baths with control strips.

## Results

### General observations

All porcine ureteral strips (mean weight, 0.0323 ± 0.0021 g, *n* = 132) were allowed to equilibrate to a passive tension of 1.53 g ± 0.15 g. During the equilibration period, none of the distal ureteral strips developed spontaneous contractions where they all remained quiescent in the absence of agonists. When quiescent tissues were subjected to phenylephrine (300 μM) or 5-HT (100 μM), they developed bursts of phasic contractions, as seen in Fig. 1. Increasing concentrations of phenylephrine (100 nM–1 mM) and 5-HT (300 nM–300 μM) resulted in increase in frequency and general phasic contractility measured as area under the curve (AUC) in a concentration-dependent manner. The amplitude of these phasic contractions was not agonist concentration-dependent and thus, expressed as maximum amplitude. Phenylephrine (300 μM) and 5-HT (100 μM) resulted in phasic contractions that had similar maximum amplitude values (phenylephrine, 1.91 ± 0.18 N/g; 5-HT, 1.81 ± 0.20 N/g).

### Relaxatory effects of mirabegron on ureteral contractility

**Fig. 2:**
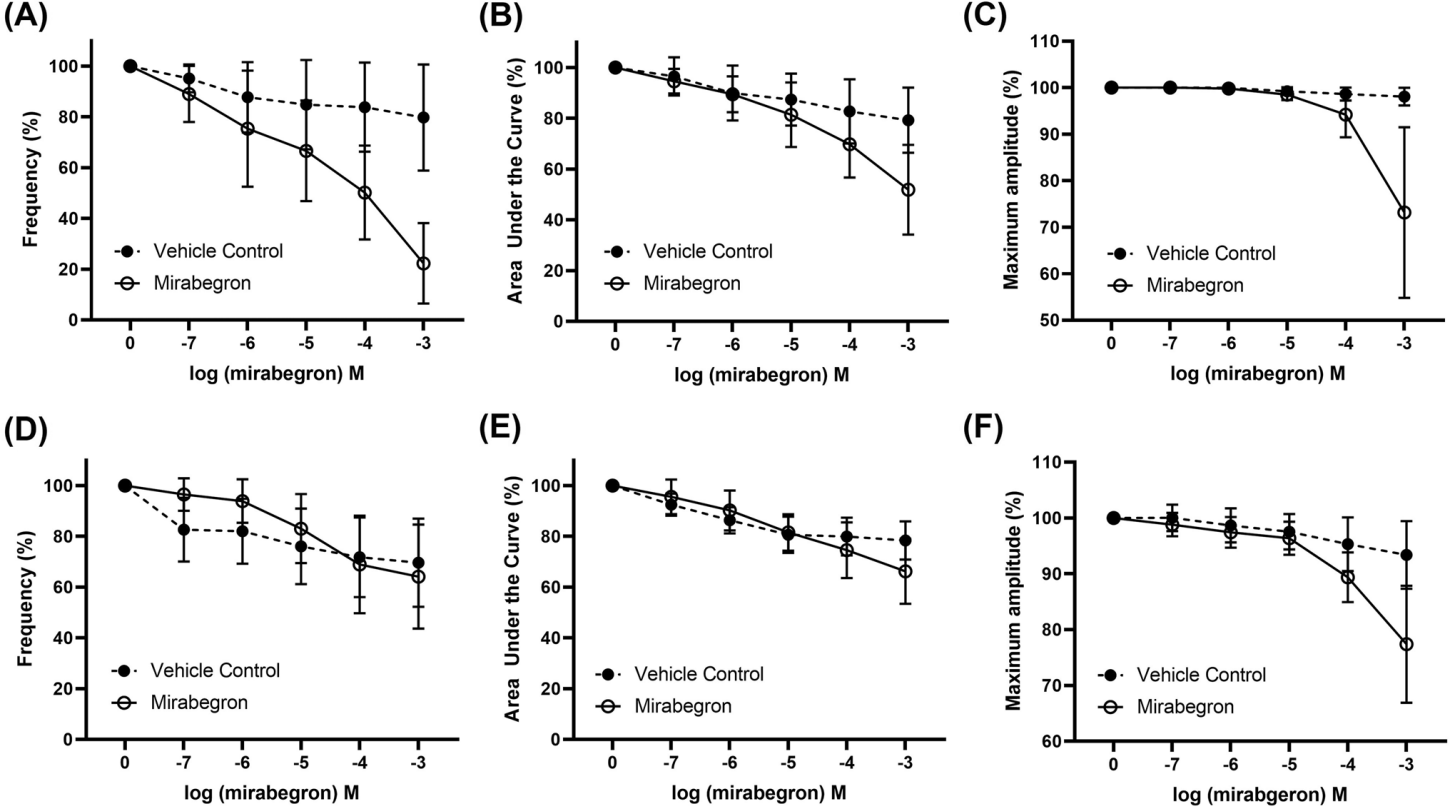
Concentration–response to mirabegron (hollow circles, solid lines) in porcine isolated distal ureteral tissue vs paired time and vehicle control (filled circles, dotted lines). Reduction in contractility was calculated relative to the initial contraction produced by **A**–**C** phenylephrine (300 µM) and **D**–**F** 5-HT (100 µM) in each tissue strip. Results are presented as mean ± SD (*n* = 6–8)

**Fig. 3:**
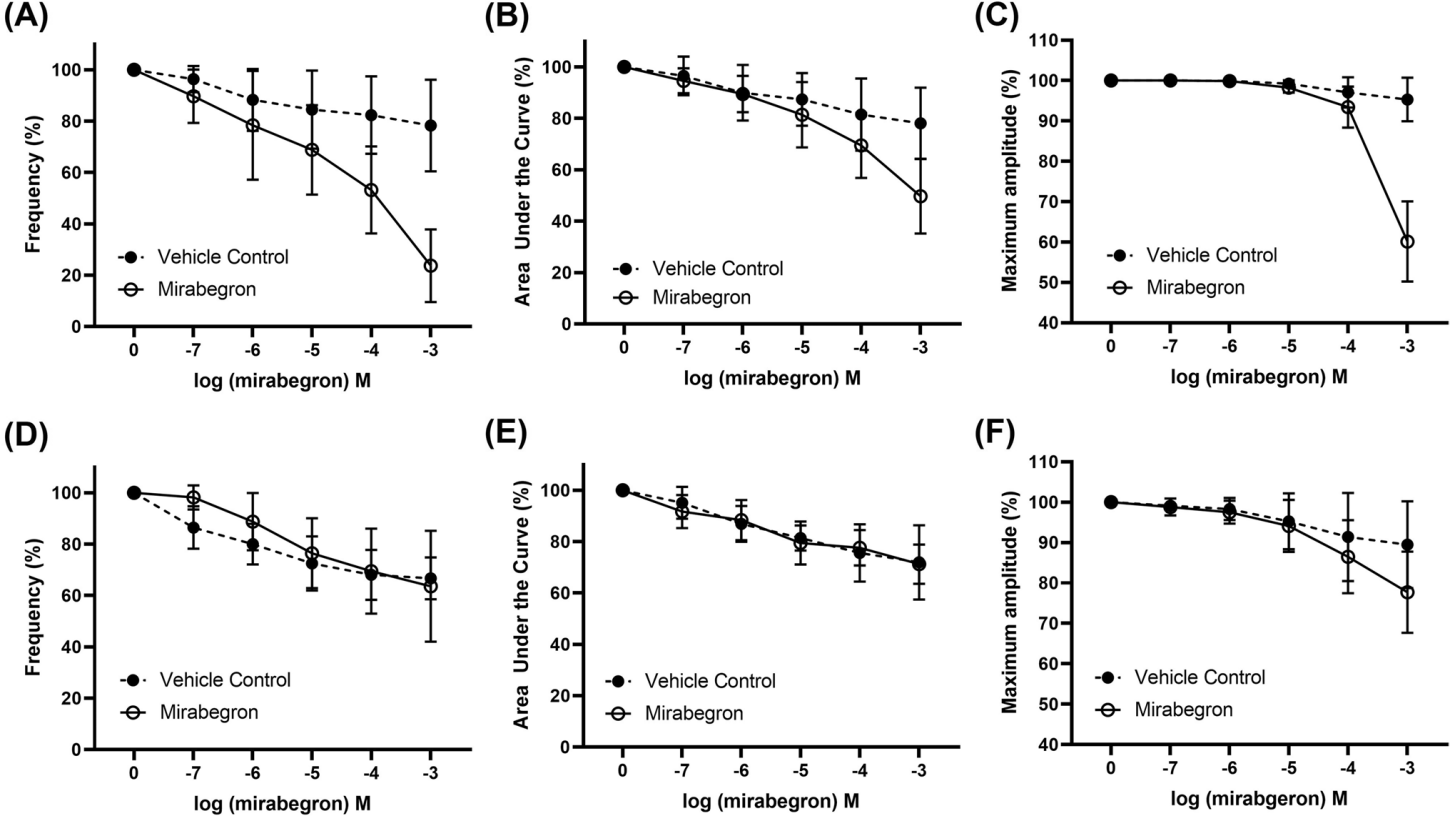
Concentration–response to mirabegron (hollow circles, solid lines) in porcine isolated distal ureteral tissue vs paired time and vehicle control (filled circles, dotted lines) in the presence of SR59230A (10 μM). Reduction in contractility was calculated relative to the initial contraction produced by **A**–**C** phenylephrine (300 µM) and **D**–**F** 5-HT (100 µM) in each tissue strip. Results are presented as mean ± SD (*n* = 7–8)

Upon generation of consistent phasic contractility induced by phenylephrine (300 μM), increasing concentrations of mirabegron (100 nM–1 mM) reduced the frequency and general contraction expressed as AUC of the contractions. However, when compared to the time and vehicle control, only high concentrations of mirabegron (10 μM and above) resulted in significant reduction of frequency (Fig. 2A), while reduction of AUC and amplitude contractions required even higher concentrations of the drug (100 μM and above, Figs. 2B–2C). When the experiment was repeated in the presence of SR 59230A (10 μM), similar findings were observed (Figs. 3A–3C). When pre-contracted with 5-HT (100 μM), mirabegron did not attenuate frequency of the ureteral contractile responses (Fig. 2D) at any concentration tested and only reduced AUC and maximum amplitude of contractile responses at 100 μM and above. Similar observations were found when these experiments were repeated in the presence of SR 59230A (10 μM) (Figs. 3D–3F).

### Effects of mirabegron on agonists-induced concentration–response

**Fig. 4:**
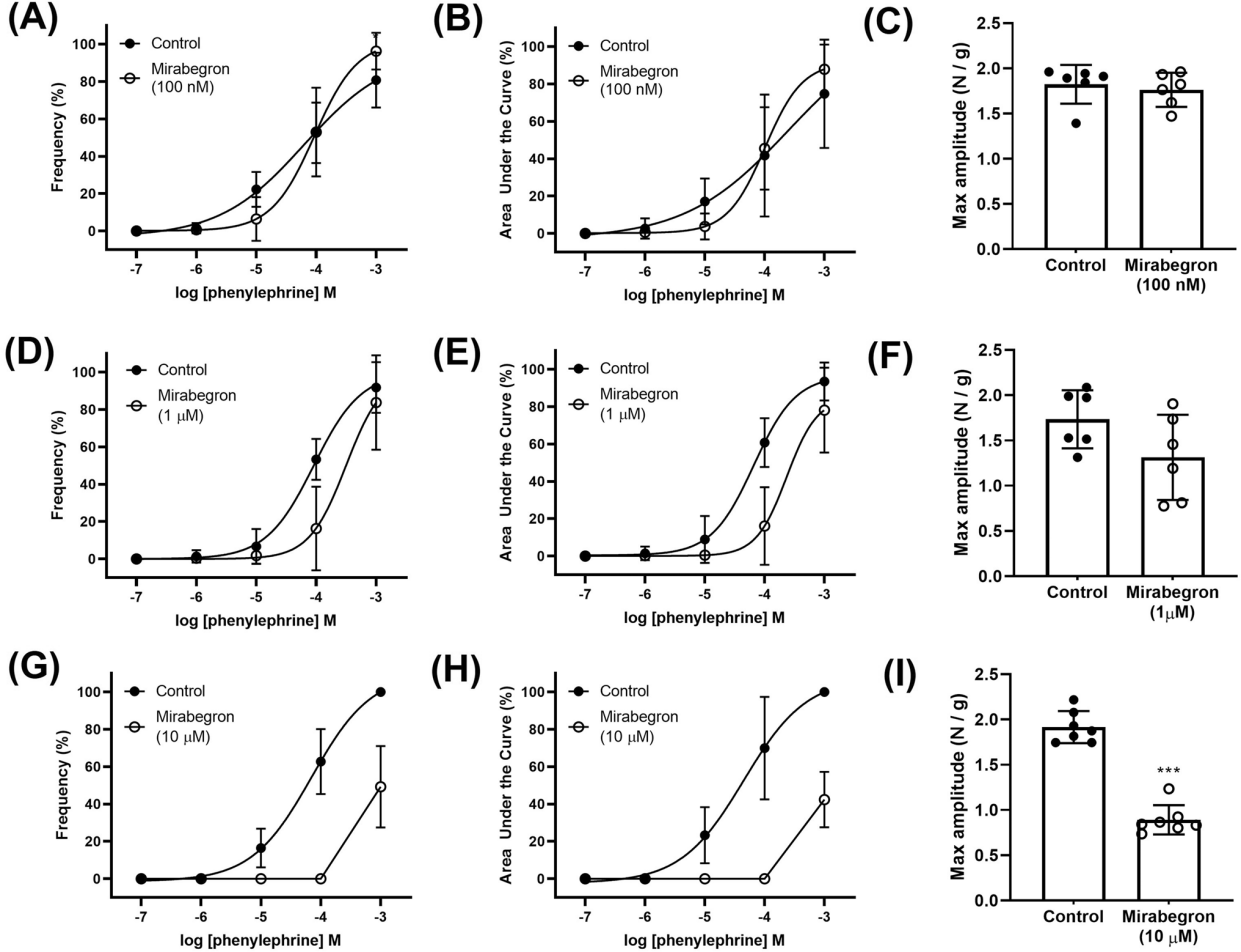
Phenylephrine-induced (100 nM–1 mM) contractions in isolated porcine distal ureter in the absence (circle) and presence (square) of mirabegron (**A**–**C** 100 nM, **D**–**F** 1 μM, **G**–**I** 10 μM). Concentration–response curves were expressed in frequency (**A**, **D**, **G**) and AUC (**B**, **E**, **H**). Results are presented as mean ± SD (*n* = 6–8)

**Fig. 5:**
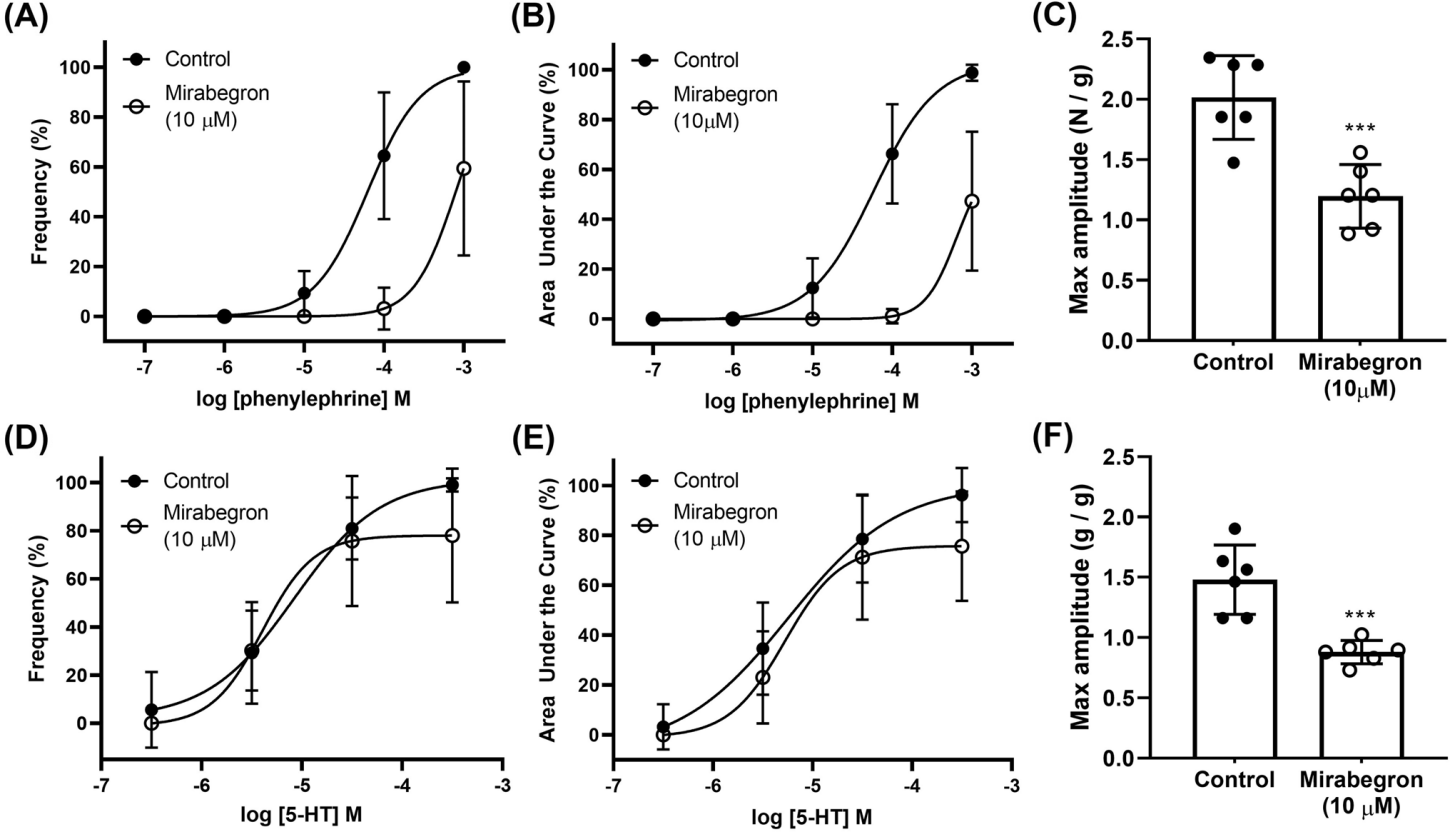
Phenylephrine-induced (100 nM–1 mM, **A**–**C**) and 5-HT-induced (300 nM–300 μM, **D**–**E**) contractions in porcine isolated distal ureteral in the absence (circle) and presence (square) of mirabegron. Phenylephrine concentration responses were performed in the presence of SR 59230A (10 μM, **A**–**C**). Concentration–response curves were expressed in frequency (**A**, **D**, **G**) and AUC (**B**, **E**, **H**). Results are presented as mean ± SD (*n* = 7–8)

Mirabegron, at 1 μM and 10 μM, produced a rightward shift of the phenylephrine concentration–response curves expressed as frequency (1 μM, *p* = 0.0351; 10 μM, *p* < 0.0001) and AUC (1 μM, *p* = 0.0036; 10 μM, *p* < 0.0001) in the porcine distal ureteral tissues (Figs. 4D–4E, 4G–4H, Table 1). Lower concentration of mirabegron (100 nM) was not capable of producing this shift in the phenylephrine concentration–response curve. Mirabegron (10 μM) did not shift the 5-HT concentration–response curves expressed as frequency and AUC (Figs. 5D–5E). The maximum frequency, AUC and amplitude of phenylephrine- and 5-HT-induced contractile responses were reduced by mirabegron, at 10 μM (Figs. 4G–4I, 5D–5F).

**Table 1:** Mirabegron effects on the potency (pEC_50_) of phenylephrine. Results are presented as mean ± SD of ‘*n*’ preparations (paired Student’s *t* tests, ^a^*p* < 0.05 vs control)

Concentration	Frequency response			AUC response		*n*
	Control	Mirabegron	Control		Mirabegron	
100 nM	4.10 ± 0.77	4.03 ± 1.11	4.16 ± 0.40		4.15 ± 0.69	7
1 μM	4.14 ± 0.51	3.60 ± 0.49^a^	4.18 ± 0.51		3.63 ± 0.37^a^	6
10 μM	4.15 ± 0.61	3.07 ± 0.56*^a^	4.20 ± 0.40		3.07 ± 0.93*^a^	7

^*^pEC50 values for 10 μM are estimated as the maximum responses were significantly reduced

### Mediation of mirabegron-induced effects

In the presence of β_3_-adrenoceptor antagonist SR 59230A (10 μM), the mirabegron effects on phenylephrine concentration responses were similar to that observed in the absence of the antagonist, where pEC_50_ values are significantly reduced (Table 2), and maximum frequency (*p* < 0.0001), AUC (*p* < 0.0001) and amplitude decreased in the presence of mirabegron (10 μM) (Figs. 5A–5C). Mirabegron effect was also unaffected when the experiments were repeated in the presence of β_1_- and β_2_-adrenoceptor blocker propranolol (10 μM), nitric oxide synthase inhibitor l-NNA (10 μM), and α_2_-adrenoceptor antagonist yohimbine (30 nM) (Table 2).

**Table 2:** Mirabegron (10 μM) effects on the potency (pEC50) of phenylephrine in the presence of SR 59230A (10 μM), propranolol (10 μM), l-NNA (10 μM) and yohimbine (30 nM). Results are presented as mean ± SEM of ‘*n*’ preparations (paired Student’s *t* tests, ^a^*p* < 0.05 vs control)

	Frequency response			AUC response		***n***
	Control	Mirabegron	Control		Mirabegron	
**SR 59230A (10 μM)**	4.21 ± 0.40	3.10 ± 1.11*^a^	4.25 ± 0.66		3.01 ± 1.23*^a^	7
**Propranolol (10 μM)**	4.06 ± 0.57	3.01 ± 0.99*^a^	4.11 ± 0.59		3.00 ± 0.96*^a^	8
**l-NNA (10 μM)**	4.28 ± 0.99	2.99 ± 1.30*^a^	4.29 ± 0.75		3.12 ± 0.99*^a^	8
**Yohimbine (30 nM)**	4.15 ± 0.39	3.05 ± 0.64*^a^	4.16 ± 0.56		3.01 ± 0.42^*a^	6

^*^pEC50 values are estimated as the maximum responses were significantly reduced

## Discussion and conclusions

While detrusor and urethral tissue strips from the same species usually exhibit tonic contractions in response to common agonist drugs (Folasire et al. 2017; Kang et al. 2015), studies from our laboratory on the ureter have reported on the complexity of the contractile activity in this tissue (Lim et al. 2020). In this study, we report on all the components of this phasic activity, including frequency, amplitude and AUC, to ensure we can capture the complexity of the response from this tissue. Because the ureter mainly receives adrenergic stimulation, it is hypothesised that contractile activity stimulated by adrenoceptor agonists is more relevant physiologically, particularly in ureteral peristalsis (Zaitouna et al. 2017). The present study investigated the effects of mirabegron on ureteral contraction and the mechanisms associated. The results of this study suggest that in the ureter, (a) mirabegron, at β_3_-adrenoceptor selective concentrations, does not relax the tissue, (b) mirabegron, at 1 μM and higher, produces a rightward shift of phenylephrine concentration–response curve, and (c) this mirabegron-induced rightward shift does not involve β-adrenoceptors, α_2_-adrenoceptors or nitric oxide production.

When pre-contracted with agonists, mirabegron at low concentrations where it selectively activates β_3_-adrenoceptor (Igawa and Michel, 2013) did not produce significant relaxatory effects on the ureter. Although previous studies have indicated that this receptor subtype is present in the human ureter and has the potential to be a significant target for medical expulsive therapy (Matsumoto et al. 2013; Shen et al. 2017), our current findings suggest otherwise. Binding assays suggest *K*_i_ value of 2.5 nM for human β_3_-adrenoceptors, if determined in Chinese Hamster Ovary cells stably addressing human β-adrenoceptor (Tasler et al. 2012). Nevertheless, mirabegron, at concentrations of 1uM and higher, was shown to reduce the frequency, AUC and amplitude of these phenylephrine-induced contractions. Additionally, when we pre-contracted the tissues with a different agent (5-HT), this attenuation effect was not visible. Therefore, we hypothesised that the mirabegron-induced ureteral relaxations were due, at least partly, to α_1_-antagonism as this has been proposed in other tissues of the urinary tract (Michel 2020).

Our agonist concentration response studies showed that the presence of mirabegron, at 1 μM and higher, was capable of antagonising phenylephrine-induced contractility. This is not surprising, as previous studies have reported that similar concentrations of mirabegron antagonises other receptors including α_1_-adrenoceptor stimulation in the urethra (Alexandre et al. 2016) and prostate (Alexandre et al. 2016; Huang et al. 2021), and muscarinic receptors in the pig and human detrusor (Maki et al. 2019; Yamada et al. 2021). Additionally, during its regulatory submission, it was also indicated that this drug potentially antagonises α_1_-adrenoceptors (U.S. Food and Drug Administration 2012). In our ureteral study, mirabegron, at 10 μM, also decreased maximum contractions which was not observed in the urethra (Alexandre et al. 2016) or prostate (Huang et al. 2021). Due to this decrease in maximum contractions, we could only calculate apparent pA_2_ values using shifts at the lower part of the concentration curves. The apparent pA_2_ values were calculated using the rightward shifts of the phenylephrine curves in the presence of 1 and 10 μM range from 6.1 to 6.5. This affinity estimate is comparable with values reported from radioligand binding studies with human recombinant α1A- and α1D-adrenoceptors which was 6.0 (Alexandre et al. 2016). The *K*_i_ values of mirabegron for α_1_-adrenoceptor subtypes from competition assays indicated that binding affinities from high to low are as follows: *α*_1A_ (0.437 μM) > *α*_1D_ (1.8 μM) > *α*_1B_ (26 μM) (Alexandre et al. 2016). In line with our suggestion, α_1A_-adrenoceptor is known to be the functionally predominant subtype for α_1_-adrenergic contractions in the human ureter (Sasaki et al. 2011).

Although the involvement of β_3_-adrenoceptors at this point appeared unlikely, we confirmed that the β-adrenoceptor antagonist SR 59230A did not abolish this mirabegron effect. A previous study on the human atrial tissue showed that mirabegron could activate β_1_-adrenoceptors via an indirect mechanism (Mo et al. 2017). To confirm that the other β-adrenoceptor subtypes and α_2_-adrenoceptors are not involved, we also repeated the experiments in the presence of propranolol and yohimbine. Our findings suggest these receptors are not involved. Additionally, β_3_-adrenoceptor activation by mirabegron in the bladder has been shown to increase nitric oxide production to relax the detrusor muscle (Imran et al., 2013). To further cement that we do not believe this receptor and mechanism is involved, we demonstrated that the nitric oxide synthase inhibitor l-NNA did not alter the mirabegron-induced effects.

We also investigated whether mirabegron antagonises 5-HT concentration responses. This was not evident from our findings, which further suggests that the effects of mirabegron in our experiments were largely limited to α_1_-adrenoceptor antagonism. Interestingly, mirabegron (10 μM) reduced the maximum frequency, AUC and amplitude of contractile responses to 5-HT. We believe that at high concentration of 5-HT, α_1_-adrenoceptors are also activated to stimulate contraction of the ureteral smooth muscle, which was attenuated by the presence of mirabegron. This likely occurrence is supported by previous studies which have shown that α_1_-antagonists can inhibit 5-HT-induced contractile responses in the bladder (Sakai et al. 2013).

We chose to perform all studies on the distal ureter as this is one of the most common sites of stone lodgement (El-Barky et al. 2014). It will be interesting to investigate if similar effects of mirabegron are present in the proximal ureter in future studies, considering it is further anatomically from the lower urinary tract. Additionally, previous studies have shown that the proximal and distal sections of the ureter respond pharmacologically differently (Lim et al. 2020), and that receptor distribution changes throughout the length of the ureter (Itoh et al. 2007). Furthermore, previous studies that have reported a relaxatory effect of β_3_-adrenoceptor activation by mirabegron in smooth muscles of the urinary tract have mainly addressed contractions that are tonic in nature (Svalo et al. 2013; Takasu et al. 2007) and the role of β-adrenoceptors in phasic contractions is still underrepresented in the literature.

While there has been recent interest in trialling mirabegron as a medical expulsive therapy drug, our findings in this report suggest that the therapeutic doses utilised clinically, where maximal plasma level is approximately 83–167 nM (Krauwinkel et al. 2012), may not cause a relaxation of the ureter to improve stone passage. While we acknowledge that this should be investigated in human tissues for further validity, we suggest that the effects of mirabegron are limited to off-target effects that require high concentrations and β_3_-adrenoceptors might not be a significant functional target for medical expulsive therapy.

## Data Availability

Raw data has been made available.

## References

[CR1] Alexandre EC, Kiguti LR, Calmasini FB, Silva FH, da Silva KP, Ferreira R (2016). Mirabegron relaxes urethral smooth muscle by a dual mechanism involving beta3 -adrenoceptor activation and alpha1 -adrenoceptor blockade. Br J Pharmacol.

[CR2] Assimos D, Krambeck A, Miller NL, Monga M, Murad MH, Nelson CP (2016). Surgical Management of Stones: American Urological Association/Endourological Society Guideline, PART I. J Urol.

[CR3] Campschroer T, Zhu XY, Vernooij RWM, Lock TMTW (2018). alpha-blockers as medical expulsive therapy for ureteric stones: a Cochrane systematic review. Bju Int.

[CR4] El-Barky E, Ali Y, Sahsah M, Terra AA, Kehinde EO (2014). Site of impaction of ureteric calculi requiring surgical intervention. Urolithiasis.

[CR5] Folasire OS, Chess-Williams R, Sellers DJ (2017). Inhibitory effect of the urothelium/lamina propria on female porcine urethral contractility & effect of age. Clin Exp Pharmacol Physiol.

[CR6] Forman A, Andersson KE, Henriksson L, Rud T, Ulmsten U (1978). Effects of nifedipine on the smooth muscle of the human urinary tract in vitro and in vivo. Acta Pharmacol Toxicol (copenh).

[CR7] Gillams K, Juliebo-Jones P, Juliebo SO, Somani BK (2021). Gender differences in kidney stone disease (KSD): findings from a systematic review. Curr Urol Rep.

[CR8] Huang R, Liu Y, Ciotkowska A, Tamalunas A, Waidelich R, Strittmatter F (2021). Concentration-dependent alpha1-adrenoceptor antagonism and inhibition of neurogenic smooth muscle contraction by mirabegron in the human prostate. Front Pharmacol.

[CR9] Igawa Y, Michel MC (2013). Pharmacological profile of beta3-adrenoceptor agonists in clinical development for the treatment of overactive bladder syndrome. Naunyn Schmiedebergs Arch Pharmacol.

[CR10] Imran M, Najmi AK, Tabrez S (2013). Mirabegron for overactive bladder: a novel, first-in-class beta3-agonist therapy. Urol J.

[CR11] Itoh Y, Kojima Y, Yasui T, Tozawa K, Sasaki S, Kohri K (2007). Examination of alpha 1 adrenoceptor subtypes in the human ureter. Int J Urol.

[CR12] Kang SH, McDermott C, Farr S, Chess-Williams R (2015). Enhanced urothelial ATP release and contraction following intravesical treatment with the cytotoxic drug, doxorubicin. Naunyn Schmiedebergs Arch Pharmacol.

[CR13] Krauwinkel W, van Dijk J, Schaddelee M, Eltink C, Meijer J, Strabach G (2012). Pharmacokinetic properties of mirabegron, a beta3-adrenoceptor agonist: results from two phase I, randomized, multiple-dose studies in healthy young and elderly men and women. Clin Ther.

[CR14] Lim I, Chess-Williams R, Sellers D (2020). A porcine model of ureteral contractile activity Influences of age, tissue orientation, region, urothelium, COX and NO. J Pharmacol Toxicol Methods.

[CR15] Maki T, Kajioka S, Itsumi M, Kareman E, Lee K, Shiota M (2019). Mirabegron induces relaxant effects via cAMP signaling-dependent and -independent pathways in detrusor smooth muscle. Low Urin Tract Symptoms.

[CR16] Matsumoto R, Otsuka A, Suzuki T, Shinbo H, Mizuno T, Kurita Y (2013). Expression and functional role of beta3 -adrenoceptors in the human ureter. Int J Urol.

[CR17] Michel MC (2020). alpha1-adrenoceptor activity of beta-adrenoceptor ligands - An expected drug property with limited clinical relevance. Eur J Pharmacol.

[CR18] Michel MC, Murphy TJ, Motulsky HJ (2020). New author guidelines for displaying data and reporting data analysis and statistical methods in experimental biology. J Pharmacol Exp Ther.

[CR19] Mo W, Michel MC, Lee XW, Kaumann AJ, Molenaar P (2017). The beta3 -adrenoceptor agonist mirabegron increases human atrial force through beta1 -adrenoceptors: an indirect mechanism?. Br J Pharmacol.

[CR20] Sakai T, Kasahara K, Tomita K, Ikegaki I, Kuriyama H (2013). Naftopidil inhibits 5-hydroxytryptamine-induced bladder contraction in rats. Eur J Pharmacol.

[CR21] Sasaki S, Tomiyama Y, Kobayashi S, Kojima Y, Kubota Y, Kohri K (2011). Characterization of alpha1-adrenoceptor subtypes mediating contraction in human isolated ureters. Urology.

[CR22] Shen H, Chen Z, Mokhtar AD, Bi X, Wu G, Gong S (2017). Expression of beta-adrenergic receptor subtypes in human normal and dilated ureter. Int Urol Nephrol.

[CR23] Svalo J, Nordling J, Bouchelouche K, Andersson KE, Korstanje C, Bouchelouche P (2013). The novel beta3-adrenoceptor agonist mirabegron reduces carbachol-induced contractile activity in detrusor tissue from patients with bladder outflow obstruction with or without detrusor overactivity. Eur J Pharmacol.

[CR24] Takahashi S, Mishima Y, Kuroishi K, Ukai M (2022). Efficacy of mirabegron, a beta3 -adrenoreceptor agonist, in Japanese women with overactive bladder and either urgency urinary incontinence or mixed urinary incontinence: post-hoc analysis of pooled data from two randomized, placebo-controlled, double-blind studies. Int J Urol.

[CR25] Takasu T, Ukai M, Sato S, Matsui T, Nagase I, Maruyama T (2007). Effect of (R)-2-(2-aminothiazol-4-yl)-4'-{2-[(2-hydroxy-2-phenylethyl)amino]ethyl} acetanilide (YM178), a novel selective beta3-adrenoceptor agonist, on bladder function. J Pharmacol Exp Ther.

[CR26] Tang QL, Wang DJ, Zhou S, Tao RZ (2021). Mirabegron in medical expulsive therapy for distal ureteral stones: a prospective, randomized, controlled study. World J Urol.

[CR27] Tasler S, Baumgartner R, Behr-Roussel D, Oger-Roussel S, Gorny D, Giuliano F, Ney P (2012). An aryloxypropanolamine hβ3-adrenoceptor agonist as bladder smooth muscle relaxant. Eur J Pharm Sci.

[CR28] U.S. Food and Drug Administration (2012) Full prescribing information for MYRBETRIQ. https://www.accessdata.fda.gov/drugsatfda_docs/label/2018/202611s011lbl.pdf

[CR29] Yamada S, Chimoto J, Shiho M, Okura T, Morikawa K, Wakuda H (2021). Possible involvement of muscarinic receptor blockade in mirabegron therapy for patients with overactive bladder. J Pharmacol Exp Ther.

[CR30] Zaitouna M, Alsaid B, Lebacle C, Timoh KN, Benoit G, Bessede T (2017). Origin and nature of pelvic ureter innervation. Neurourol Urodyn.

